# Characteristics of fetal physiological and pathological uterine effusion observed on prenatal ultrasonography: a case report

**DOI:** 10.1186/s12884-022-04715-x

**Published:** 2022-05-12

**Authors:** Lei Wang, Lizhu Chen, Dongmei Li, Bing Wang, Zeyu Yang

**Affiliations:** 1 Department of Ultrasound, Shengjing Hospital of China Medical University, No. 36 Sanhao St, Shenyang, 110004 Liaoning China; 2 Department of Ultrasound, Shenyang Women’s and Children’s Hospital, Shenyang, Liaoning China

**Keywords:** Fetal uterine effusion, Hydrometrocolpos, Ultrasonography, Imperforate hymen

## Abstract

**Background:**

The prenatal detection rate of fetal uterine effusion is very low, and current case reports mainly focus on pathological hydrometrocolpos. We presented two cases of fetal physiological uterine effusion with different ultrasonic characteristics and compared them with one case of hydrometrocolpos with the hope of identifying strategies to reduce misdiagnosis of fetal uterine effusion.

**Case presentation:**

This paper reports the cases of two female fetuses with abnormal pelvic echoes in the third trimester, referred to a tertiary center to be screened for suspected pelvic teratoma and cystic mass, respectively. Ultrasound consultation revealed fetal uterine effusion. The two fetuses were delivered at our hospital after a full term. Re-examining the uterus and adnexa of the neonates revealed that the uterine effusion had subsided naturally. Another female fetus had a large cystic mass in the pelvic cavity in the third trimester, and prenatal examination indicated fetal hydrometrocolpos. The fetus was delivered at our hospital after a full term. The hydrometrocolpos existed even after birth. After consultation with a neonatal surgeon and gynecologist, the newborn was diagnosed with congenital imperforate hymen with hydrometrocolpos. Hymen puncture and open drainage led to a good prognosis.

**Conclusions:**

Prenatal ultrasonography plays an important role in diagnosing and differentiating between physiological and pathological fetal uterine effusion. It can help reduce misdiagnoses that can lead to incorrect clinical decisions.

## Background

Fetal internal genitalia are often not assessed in prenatal fetal malformation screening. Only in cases with related family history, difficulty distinguishing the external genitalia, and gender-related conditions, the focus is on internal genitalia scanning [1, 2]. The fetal uterus is located between the bladder and rectum, and the myometrium of the uterus is moderately echoic, similar to the intestinal or bladder wall; consequently, it is challenging to distinguish the uterus from the surrounding tissues. Due to the prenatal changes in the uterus, there are chances of misdiagnosis in female fetuses [3]. Our previous cohort study measured the anterior-posterior and transverse diameters of the fetal uterus at different gestational weeks, and the fetal uterus could be visualized from the 19th gestational week, this observation is consistent with a previous report [4].

A few cases of pathological fetal hydrometrocolpos caused by abnormalities in the urogenital tract or cloaca have been reported in the literature; however, there are no reports on fetal physiological uterine effusion [5]. We present three cases of uterine effusion and discuss the features appearing on the ultrasound to differentiate physiological and pathological causes. Even if it may not be feasible to soundly diagnose a physiological uterine effusion prenatally, this difference in prenatal ultrasonography suggests that further assessments are needed postnatally to confirm spontaneous absorption.

## Case presentation

### Case 1

**Fig. 1:**
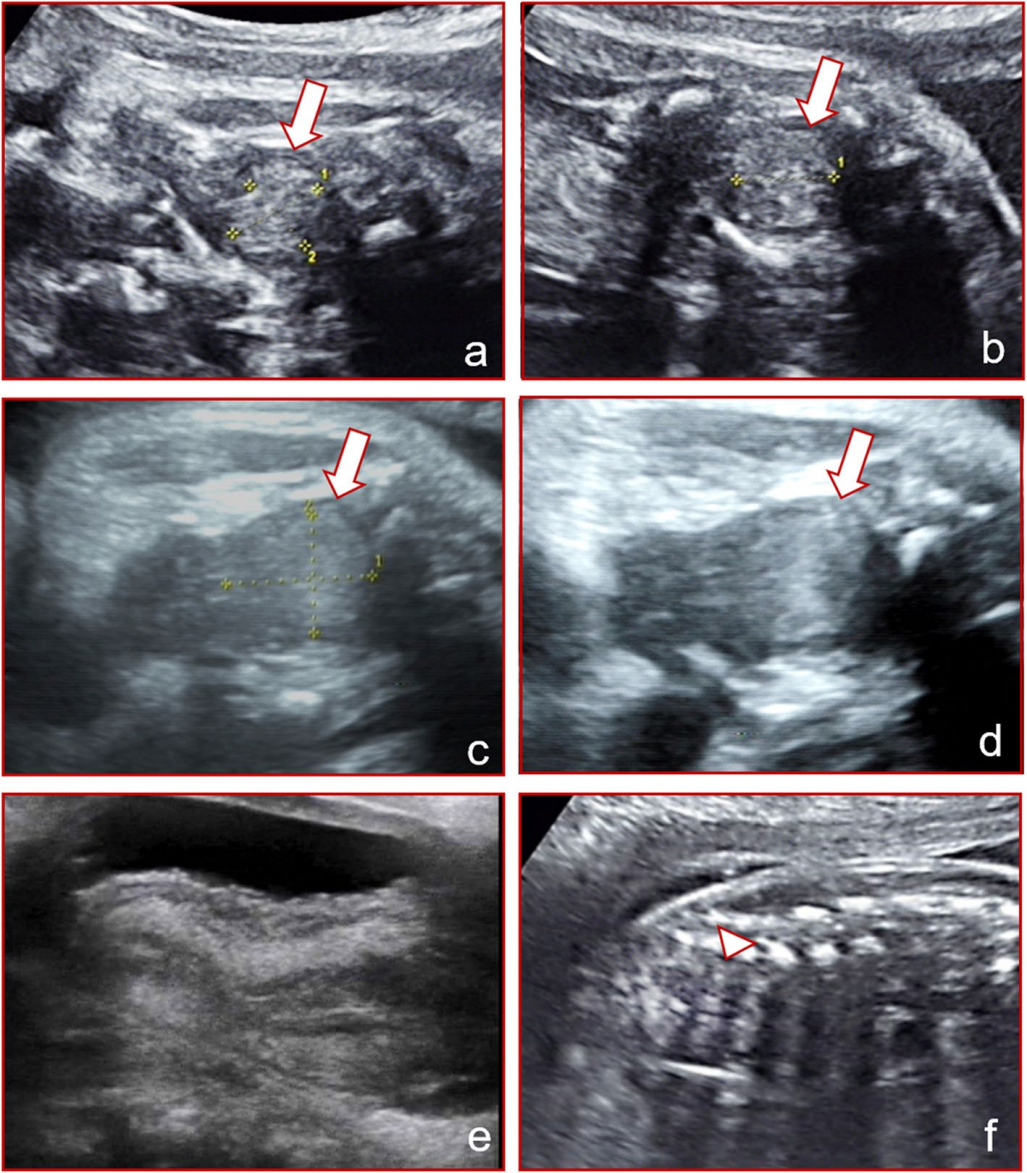
Ultrasonographic comparison between the case of uterine effusion showing a hyperechoic mass (*arrow*) and sacrococcygeal teratoma (*arrowhead*): prenatal images of uterine effusion in different sections and gestational weeks (**a** to **d**); postnatal image of the uterus (**e**); ultrasound image in another case of sacrococcygeal teratoma at 24 weeks of gestation for contrast (**f**), the diagnosis was confirmed by postoperative pathology

A 30-year-old woman (gravida 1 para 0) was referred for a hyperechoic mass in the pelvic cavity of the female fetus at 28 weeks gestation. A magnetic resonance imaging (MRI) showed features suggestive of a teratoma. She was subsequently referred to our center at 31 weeks gestation. According to our previous data, the mean expected fetal uterine anterior-posterior diameter at 31 weeks gestation is 8.73 ± 0.86 mm. Our ultrasound examination revealed a hyperechoic mass in the fetal pelvic cavity measuring 1.5 × 1.5 × 1.3 cm. The mass was located between the bladder and rectum, surrounded by myometrium (Fig. 1a and b). No significant blood flow signals were detected on color doppler flow imaging (CDFI). Normal female external genitalia were visible. We planned to reassess the neonate at birth. High-throughput sequencing analysis of fetal free DNA in maternal peripheral blood was normal, and the fetal chromosome karyotype was 46XX. Ultrasound examination at 34^+ 4^ weeks showed a slightly larger pelvic mass, measuring 2.3 × 2.2 × 1.7 cm (Fig. 1c and d). The patient had a spontaneous vaginal delivery at 39 weeks gestation and delivered a healthy neonate. The external genitalia appeared normal. An abdominal ultrasound was performed on the second day after birth, however, the pelvic mass could not be visualized (Fig. 1e). Hence, we concluded that this was a physiological uterine effusion rather than a teratoma (shown in Fig. 1f with a contrast).

### Case 2

**Fig. 2:**
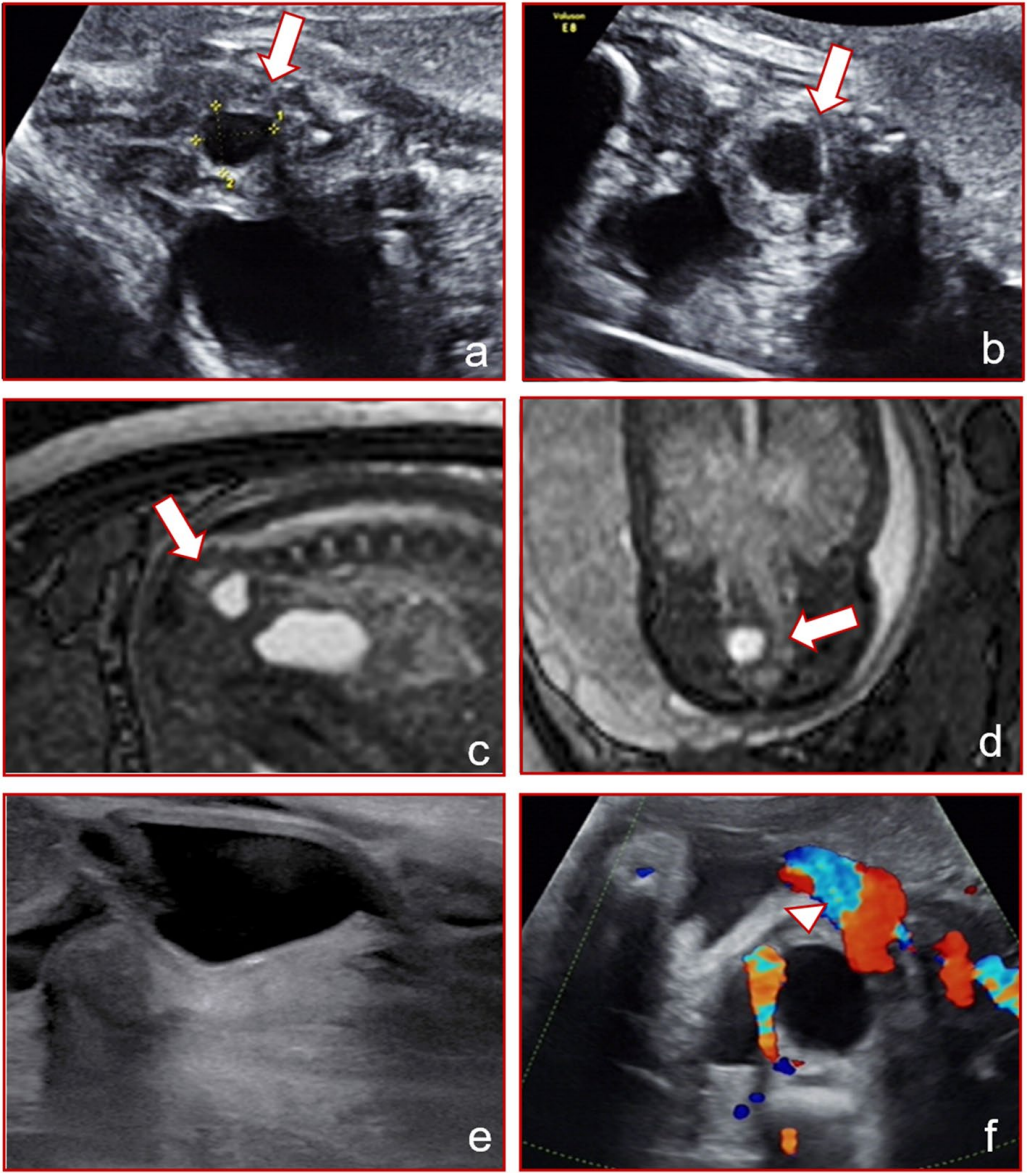
Comparison between the case of uterine effusion showing an anechoic cyst (*arrow*) and ovarian cyst (*arrowhead*): ultrasound imaging of uterine effusion in different sections at 28^+ 2^ weeks of gestation (**a** and **b**); MRI images of uterine effusion in different sections at 28^+ 2^ weeks of gestation (**c** and **d**); postnatal image of the uterus (e); ultrasound image in another case of ovarian cyst at 31 weeks of gestation for contrast (f), the diagnosis was confirmed by postnatal ultrasonography

A 29-year-old woman (gravida 1 para 0) was diagnosed with a cystic lesion measuring 1.3 × 1.0 × 1.0 cm in the pelvic cavity on routine ultrasound examination at our center at 28^+ 2^ weeks gestation. According to our previous data, the mean expected fetal uterine anterior-posterior diameter at 28 weeks gestation is 7.90 ± 0.75 mm. The cystic lesion between the fetal bladder and rectum was anechoic, with a clear boundary and a myometrium-like hypoechoic layer around (Fig. 2a and b). The external genitalia appeared normal. An MRI confirmed the cystic pelvic mass, which was more to the right, measuring 1.2 × 1.0 × 1.2 cm (Fig. 2c and d). The patient had a spontaneous vaginal delivery at 40 weeks gestation and delivered a healthy neonate. The external genitalia were normal. The pelvic mass was not visible on a repeat ultrasound examination performed the next day (Fig. 2e). We concluded that this was a physiological uterine effusion, different from a fetal ovarian cyst (shown in Fig. 2f).

### Case 3

A 29-year-old woman (gravida 1 para 0) was diagnosed with fetal megacolon on routine ultrasound examination at 33^+ 5^ weeks gestation. According to our previous data, the mean expected fetal uterine anterior-posterior diameter at 34 weeks gestation is 9.93 ± 0.69 mm. Cell-free fetal DNA test results were normal, and the fetal chromosome karyotype was 46XX.

**Fig. 3:**
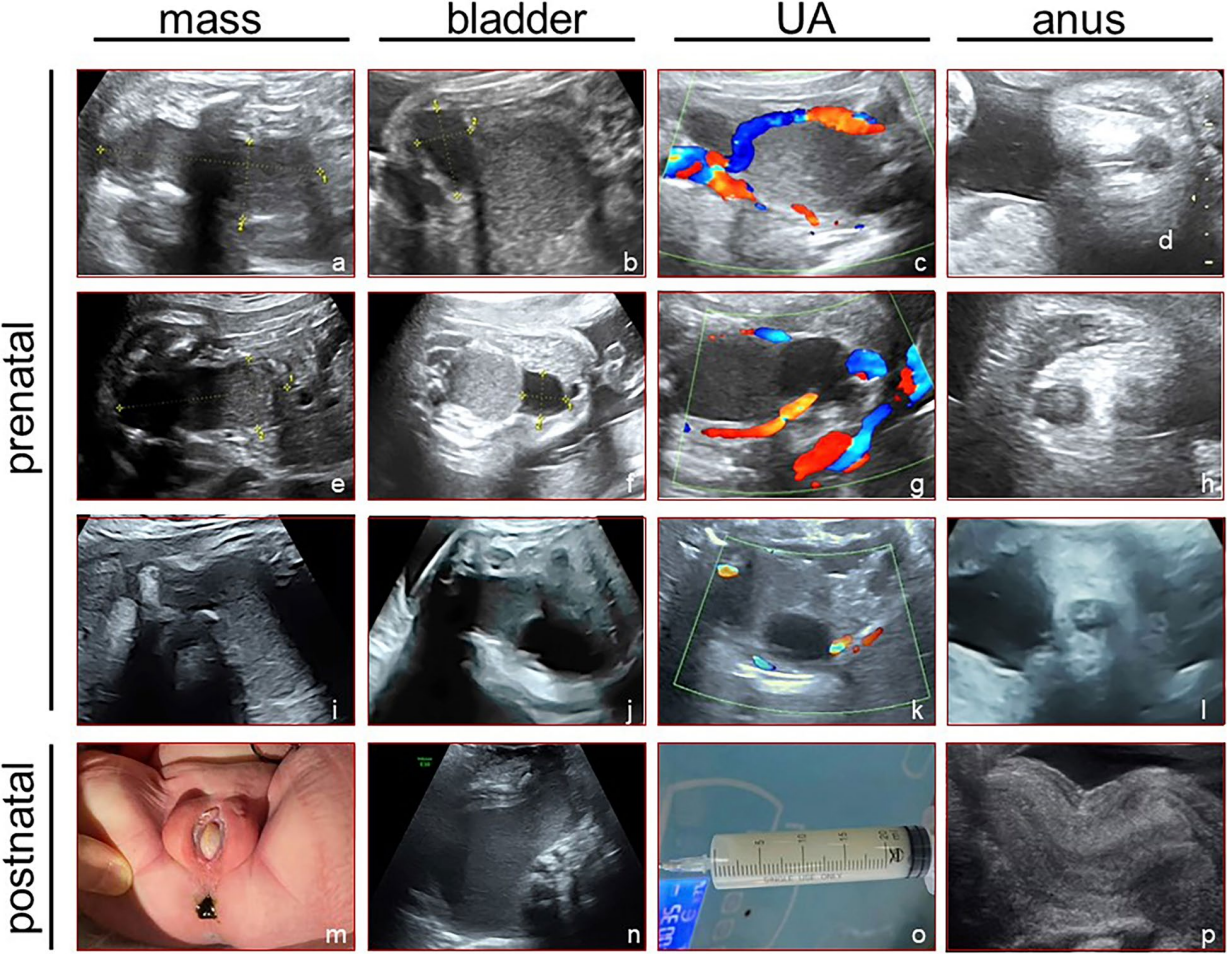
Prenatal and postnatal ultrasound findings of congenital imperforate hymen. Images arrayed in the first to fourth columns represent the ultrasound findings of mass, bladder, UA (umbilical artery), and anus in different gestational weeks. Prenatal ultrasound images: images arrayed in the first to the third row represent the ultrasound findings of 34, 34^+ 5^, and 38 weeks of gestation respectively (**a** to **l**); Postnatal observations: perineum and anus of the newborn (**m**), ultrasonography at 1 day after birth (**n**), open drainage of hydrometrocolpos (**o**), ultrasonography at 3 days after treatment (**p**)

Ultrasonography at 34 weeks of gestation showed two cystic masses in the lower abdominal cavity: the posterior inferior mass measuring 8.5 × 3.6 × 2.8 cm was filled with liquid and showed dense echogenic dots, umbilical arteries were found on both sides of the mass; the anterosuperior mass measuring 2.3 × 1.3 × 1.6 cm was closer to the anterior abdominal wall, filled with liquid. The anal “target sign” was visible [6] (Fig. 3 a–d). The amniotic fluid volume was normal. At the time of the investigation, the larger mass was considered the bladder based on its location and relationship with the umbilical arteries; however, the nature of the smaller mass was unclear.

At 34^+ 5^ weeks of pregnancy, ultrasonography showed that the posterior larger cystic mass was 7.9 × 3.4 × 3.1 cm in size, and there was no significant change in one-hour dynamic observation. The anterior smaller mass could not be observed at the start of the investigation. After dynamic observation for 30 minutes, this mass was found to be approximately 4.4 × 3.5 × 2.3 cm in size, and at 1 hour, it was approximately 2.3 × 2.3 × 2.2 cm in size. The umbilical arteries could be observed beside both the cystic masses. The anal “target sign” could be seen (Fig. 3 e-h). On this occasion, the larger mass was considered intestinal dilatation, and the smaller one was considered the bladder.

Ultrasonography at 38 weeks of pregnancy revealed that the fetal bladder was pushed upward, and the larger cystic mass could be seen posterior to the bladder, measuring 8.4 × 4.8 × 3.8 cm, with a slightly thickened wall. It extended downward in a tubular shape with an arc-like bulge in the perineum. The mass was filled with liquid and showed dense echogenic dots. No significant blood flow signals were detected on CDFI. The anal “target sign” could be seen (Fig. 3 i-l). Ultrasonography findings suggested hydrometrocolpos, and postnatal re-examination was recommended.

The pregnant mother had a history of diabetes during pregnancy, and the child was delivered by cesarean section at full term (38^+ 2^ weeks). Physical examination revealed normal female external genitalia, perineal saccular white matter protruding outward, with normal urethra and anal openings (Fig. 3m). Transabdominal ultrasonography revealed a large amount of fluid in the uterine cavity and vagina, measuring 8.4 × 5.6 × 3.8 cm, accompanied by dense echogenic dots and extending up to the umbilicus. Ultrasonography findings suggested hydrometrocolpos (Fig. 3n). After MDT consultation, congenital imperforate hymen with hydrometrocolpos was diagnosed. Subsequently, hymen puncture aspiration and open drainage were performed, and approximately 60 ml of milky white liquid was drained (Fig. 3o). On physical examination 3 days after treatment, a small amount of milky white liquid was seen flowing through the vaginal opening under compression. On ultrasonography, a hypoechoic thin layer with a thickness of approximately 0.2 cm was found extending from the uterine cavity to the cervical canal (Fig. 3p). At 7 days after treatment, no effusion was found in the uterine cavity or vagina.

## Discussion and conclusions

Fetal uterine effusion is caused by estradiol stimulating the fetal uterine glands, leading to fluid secretion in the fetal uterine cavity. Estradiol is a sex hormone produced by the ovaries and placenta. Estradiol in fetuses mainly comes from the placenta. The level of estradiol in maternal serum is 100 times higher during pregnancy. Estradiol levels in fetuses also increase with the gestational age, reaching a peak of approximately 2000–6000 pg/mL at 33–36 weeks of gestation and decreasing rapidly after birth [7]. Therefore, fetal uterine effusion mostly occurs in the third trimester, with the earliest reported case diagnosed at 25 weeks [8]. All cases reported in this paper were fetuses in the third trimester, and the earliest discovery of fetal uterine cavity effusion occurred at 28 weeks. Ultrasound images of the uterine effusion may be anechoic or isoechoic due to the change in mucus concentration in the effusion [9]. When the mucus content in the effusion is low, it is translucent anechoic, similar to a cyst (case 2, Fig. 2f). When the mucus content in the effusion is high, it shows as isoechoic or hyperechoic, which is approximately solid, and as the echo is similar to that of a tumor, a misdiagnosis of a space-occupying lesion may be made (case 1, Fig. 1f). Color Doppler imaging holds great significance in differential diagnosis [10].

In the first two cases reported in this paper, the lesions were suspected to be abnormal mass in prenatal ultrasound screening and confirmed to be physiologic uterine effusion after birth. The ultrasound imaging features were analyzed retrospectively and summarized in Table 1: Myometrial echoes were found around the mass, similar to an inverted pear shape. The mass was anechoic or isoechoic, and the diameter of the mass was 1–3 cm. The most important feature was that the mass was confined to the uterine cavity, and there was no significant finding of hydrometrocolpos in the cervical canal or vagina.

**Table 1:** Summary of the characteristics of uterine effusion in the cases

Gestational Weeks (first observed)	Location of the mass	Maximum diameter (cm)	ultrasonograpy characteristics	Range	Internal blood flow	Postnatal diagnosis	Clinical management and prognosis
28	Between the bladder and the rectum	< 3	Isoechoic or hyperechoic, with myometrial echoes around	Smal, confined to the uterine cavity	undetected	physiological uterine effusion	subsides spontaneously after birth
28^+ 2^	between the bladder and the rectum	< 3	Anechoic with myometrial echoes around	Small, confined to the uterine cavity	undetected	physiological uterine effusion	subsides spontaneously after birth
33^+ 5^	Lower back of the raised bladder, in front of the rectum up to the lower abdominal cavity	> 5	filled with liquid and showed dense echogenic dots with thinner myometrial echoes around	Large, involving the cervix and vagina	undetected	iimperforate hymen with hydrometrocolpos	hymen puncture and open drainage led to good prognosis

The general range of physiological uterine effusion is relatively small. Although it increases slightly with gestational age, it will not change drastically. In the follow-up cases, the maximum diameter of physiological uterine effusion was less than 3 cm. Physiological effusion was in regular shape and restricted by the shape of the uterine cavity. Since the condition is usually detected in the late second and third trimesters, the echo of the myometrium can be recognized around the effusion, and this can be used to distinguish the condition from other pelvic lesions.

In the last case, the pathological effusion was diagnosed as megacolon by ultrasound examination before being transferred to our hospital. The cystic mass with liquid echo and dense echogenic dots appeared similar to intestinal content, while the umbilical arteries running on both sides of the mass led to the mis-consideration of the mass being the bladder. However, in reality, a large amount of uterine effusion will lift the uterus up into the lower abdominal cavity and squeeze the bladder into the lower abdominal cavity. The bladder appears as a cystic mass on the right front side and still has the umbilical artery running on both sides. During the examination at 38 weeks of pregnancy, we clearly observed the cystic mass downward in front of the rectum and arcuate in the perineum, and the huge cystic mass communicated with the cervical canal. The anal “target sign” was complete. In contrast, after continuous dynamic observation, the anechoic mass changed in size and was confirmed to be the normal bladder. Summarily we diagnosed the condition as a malformation of the reproductive system, not intestinal dilatation. After birth, the patient’s condition was diagnosed as hymen atresia. The uterus, cervix, and upper vagina differentiate and develop from the paramesonephric ducts in the tenth week of development, while the lower vagina is formed by cavitation of the urogenital sinus in the endoderm alone. Congenital imperforate hymen is caused by failure of urogenital sinus cavitation during embryonic development [11]. Therefore, it is often accompanied by abnormalities in the urinary system but rarely by abnormal development of other female reproductive organs [12]. For case 3 in this article, the urinary system was found to be normal in both prenatal and postnatal assessments.

The differentiating factor between physiological and pathological uterine effusion is that physiological uterine effusion is generally small in range and confined to the uterine cavity. In contrast, pathological uterine effusion is often large, caused by abnormalities in the urogenital tract, cloaca, or by abnormal obstruction of the reproductive tract, and accompanied by a cervical canal and vaginal effusion.

In addition, due to the homology of the above embryonic development and the relationship between the anatomical location, it is necessary to rule out that the lesions originate from the colorectal and bladder. This is done by scanning the “fetal pelvic floor” structure [13], including the urinary system and the intestinal tract. Additionally, the umbilical and mesenteric arteries serve as anatomical markers and should be carefully scanned. Furthermore, it is important to dynamically observe the changes in the size of the lesions.

Differential diagnosis of lower abdominal cystic masses in female fetuses includes fetal sacrococcygeal cystic teratoma and cysts, intestinal dilatation, ureteral cysts, persistent cloaca, and megacystis [14]. Due to the homology of embryonic development, we should also consider the possibility of fetal uterine effusion in patients with urorectal septum malformation sequence and cloacal or urogenital tract abnormalities [15].

It has been reported that there is a good correlation between prenatal ultrasound images and MRI of uterine and vaginal effusion [5]. When it is difficult to obtain clear ultrasound images, especially in the third trimester affected by fetal posture and amniotic fluid volume, MRI can be used as a supplement to ultrasound examination.

Physiological fetal uterine effusion does not require clinical treatment and subsides naturally in the neonatal period after birth. Congenital imperforate hymen requires open drainage after birth, and the prognosis is good. Pathological uterine effusion due to abnormalities such as urorectal septum malformation sequence and cloacal or urogenital tract abnormalities have different prognoses depending on the characteristics of the corresponding malformation.

From the cases described in this report, it can be concluded that the diagnosis of physiological uterine effusions may be suspected prenatally, especially if it suits the suggested ultrasonographic features elaborated and confined to the uterus. However, it still must be confirmed postnatally. Timely referral and early involvement of MDT management are recommended to reduce unnecessary intervention and parental anxiety, especially for simple physiological uterine effusions.

## Data Availability

All data generated or analyzed during this study are included in this published article.

## Abbreviations

CDFI: Magnetic resonance imaging
MDT: Multidisciplinary team
MRI: Color doppler flow imaging
